# Old Saws and Their Application (?) to Medicine

**Published:** 1915-06

**Authors:** 


### Old Saws and Their Application (?) To Medicine

"See a pin and pick it up, all the day you'll have good luck" or very bad luck in the way of tetanus, tuberculosis, sepsis, etc.

Many a life has been sacrificed for the sake of saving an old mattress, infected bedding, clothing, etc. Rummage sales have had to be forbidden by law in some places, owing to the danger of infection. Almost anyone could keep several women and children supplied with handkerchiefs by picking up lost ones on the street but it does not pay.

"A penny saved is a penny gained." But without regard to the above danger, small economies are likely to entail big neglects in practice and it is also important to count time and strength. It is a good rule to destroy anything that does not serve its purpose without causing delay and danger of failure in an emergency, or else to have it promptly repaired and, generally, by some one who makes a business of the kind of work concerned.

"If you want a thing well done, do it yourself" but, if it is something outside your proper line of work or of a lower order of work, it is a good rule to get some one else to do it for you and to insist that it shall be done at least as well as you can do it yourself.

"You cannot turn the mill-wheel with the water that is past." But, the men who make a pecuniary success of medicine are the ones that do just this. Of one such man, it was said that he had read essentially the same paper at every important medical meeting for twenty years. And, often, the mill down-stream, is far more prosperous than the one highest up that first used the water.

"You can catch more flies with molasses than with vinegar." But, taking a hundred ideally sweet and amiable physicians and a hundred surly, sour ones, we question very much whether the same difference will be found so far as catching patients and dollars is concerned, or whether the former will get any larger attendance at memorial meetings or any more eloquent obituaries from their colleagues.

